# An Immune Cell-Based Signature Associating With EMT Phenotype Predicts Postoperative Overall Survival of ESCC

**DOI:** 10.3389/fonc.2021.636479

**Published:** 2021-04-01

**Authors:** Hongliang Yu, Dayong Gu, Chao Yue, Jianhua Xu, Feng Yan, Xia He

**Affiliations:** ^1^ Department of Radiation Oncology, Affiliated Cancer Hospital of Nanjing Medical University, Jiangsu Cancer Hospital, Jiangsu Institute of Cancer Research, Nanjing, China; ^2^ Department of General Surgery, Affiliated Cancer Hospital of Nanjing Medical University, Jiangsu Cancer Hospital, Jiangsu Institute of Cancer Research, Nanjing, China; ^3^ Department of Clinical Laboratory, Affiliated Cancer Hospital of Nanjing Medical University, Jiangsu Cancer Hospital, Jiangsu Institute of Cancer Research, Nanjing, China

**Keywords:** immune signature, overall survival, esophageal squamous cell carcinoma, epithelial-mesenchymal transition, M2 macrophage

## Abstract

Esophageal squamous cell carcinoma (ESCC) is one of the deadliest solid malignancies and has a poor survival rate worldwide. In this study, we aimed to establish a tumor-infiltrating immune cell-based prognosis signature (IPS) to predict patients’ survival times and aid in the development of targeted therapies or immunotherapies. The abundances of 22 types of immune cells were determined by the CIBERSORT algorithm from ESCC patient gene expression data in the Gene Expression Omnibus (GEO) training set (n = 179) and The Cancer Genome Atlas (TCGA) validation set (n = 95). Then, the IPS was established by using the least absolute shrinkage and selection operator (LASSO) regression method. Kaplan-Meier analysis showed that patients with high IPS scores had significantly worse overall survival times than patients with low IPS scores in both the training set and the validation set (log-rank p = 0.001, and p = 0.050, respectively). Univariate and multivariate Cox regression analyses proved that the IPS was a robust prognostic factor for ESCC, independent of age, sex, tumor node metastasis (TNM) stage, pathology grade, and tumor location. In the mechanistic study, the epithelial-mesenchymal transition (EMT) process was identified by both gene set enrichment analysis (GSEA) and weighted correlation network analysis (WGCNA) as the underlying mechanism by which the IPS affects the prognosis of ESCC. After systematic correlation analyses, we found that M2 macrophages were the only cell type in the IPS significantly correlated with the EMT process. This relationship between M2 macrophage infiltration and the EMT phenotype was also confirmed by our preliminary immunochemistry (IHC) and multiplexed immunofluorescence study. In conclusion, we constructed an IPS that predicts the postoperative prognosis of ESCC patients and uncovered the critical role of M2 macrophages in the interplay between immune status and the EMT phenotype in ESCC.

## Introduction

Esophageal squamous cell carcinoma (ESCC) is one of the most common malignancies posing a severe threat to human health worldwide especially in East Asia. Despite recent improvements in diagnostics and therapeutics, the treatment outcome for ESCC remains poor, with a 5-year overall survival rate of only approximately 20% (1). There is an urgent need to better understand the prognostic factors affecting ESCC to develop novel biomarkers and targeted therapies.

With the rise of revolutionary tumor immunotherapy over the last decade, there is accumulating evidence that tumor cell-extrinsic factors also affect tumor prognosis. Many studies have shown evidence that tumor infiltrating immune cells can secrete and interact with various cytokines and chemokines, facilitate crosstalk between matrix cells and tumor cells, and play critical roles in mediating inflammation or cytotoxicity in the tumor micromilieu, acting as both pro- and antitumor effectors (2). There have been several studies focusing on the impact of immune infiltrating cells on the prognosis of ESCC (3). However, prior immunohistochemistry (IHC)-based cell biomarker studies have a common limitation: only a few routine immune cell biomarkers, most frequently CD3, CD4, CD8, CD45RO, and FoxP3, were investigated (3). Thus, these studies provide only a limited understanding of the vast immune microenvironment scenarios of ESCC. Additionally, the specificity and sensitivity with which a given cell biomarker can identify certain immune cell subtypes are still under debate (4). In addressing these questions, bioinformatics has long played a critical role (5). Many prior well-designed studies adopted bioinformatic methods and successfully revealed the relationships between the immune cell population and phenotypic traits in many types of cancers (6–9). Moreover, ongoing progress in computational methods has enabled higher accuracy in the quantification of the immune cells, greatly aiding in immune-related research of tumors (10–12).

In the present study, by applying bioinformatics methods, the abundances of 22 types of immune cells in ESCC were derived from gene expression data in two independent data sets. An immune cell-based prognostic signature (IPS) was established by the least absolute shrinkage and selection operator (LASSO) method in the training set. The reliability of the constructed IPS was then verified in the independent validation set. We also investigated the possible mechanisms by which the IPS influenced the survival prognosis of ESCC. Our preliminary results showed that the epithelial-mesenchymal transition (EMT) process is closely related to the IPS in ESCC. Additionally, after systematic correlation analyses, we found that M2 macrophages were the only immune cell type in the IPS significantly linked to the EMT process, implicating their possible critical role in the interplay between the EMT process and immune features in ESCC.

## Materials and Methods

### Data Sources and Processing

After comprehensive data searching, two independent patient cohorts from the Gene Expression Omnibus (GEO) and The Cancer Genome Atlas (TCGA) databases were enrolled in the present study. We downloaded the microarray gene expression profiles of 179 pairs of ESCC tumor tissues and adjacent normal tissues, as well as the corresponding clinicopathological information from the GEO database (http://www.ncbi.nlm.nih.gov/geoprofiles, GSE53625). TCGA RNA-seq data (in FPKM format) for 196 EC samples were downloaded from the University of California, Santa Cruz (UCSC) Xena site (https://xena.ucsc.edu). Of the 196 TCGA samples, 95 samples with a pathology type of ESCC were eligible for further research. Accompanying clinical data, such as sex, age, tumor grade, clinical stage, and survival time, were also downloaded. Then, we obtained proper matrices of gene expression data and clinical data by using R software (R Foundation for Statistical Computing, Vienna, Austria).

### Assessment of the Abundances of Tumor-Infiltrating Immune Cells

The CIBERSORT algorithm (10) was applied to evaluate tumor-infiltrating immune cell abundances in ESCC tumor samples. CIBERSORT is an analytical method designed and robustly validated to identify 22 human immune cell types from gene expression data of mixed cell populations, outperforming other methods in noise and unknown mixed content (10, 13). The p-value and root mean squared error (RMSE) were calculated for each sample. Samples with a p-value < 0.05 were enrolled for further research.

### Development of the IPS for ESCC

Logistic regression analysis was conducted with LASSO regularization as the variable selection method to identify the IPS that best predicted survival. The LASSO method is a powerful shrinkage and variable selection method for the regression of high-dimensional data (14). As LASSO requires tuning of the parameter λ, which regulates regularization strength, 10-fold cross-validation was used for λ selection in the LASSO regression model. All these processes were performed with the “glmnet” package (14) in R software.

### Identification of IPS-Associated Biological Pathways and Processes

In this study, weighted correlation network analysis (WGCNA) (15) was applied to investigate the significantly correlated gene clusters and their relationships with clinical traits in patients with high and low IPS scores. The genes were ranked by the standard deviation of individual gene expression from large to small, and we chose the top 5000 genes for WGCNA, which was performed with the “WGCNA” package in R software (16). The hierarchical clustering function was used to classify genes with similar expression patterns into clusters based on topological overlap matrix (TOM) dissimilarity with a minimum size of 50. A power of β = 12 and a scale-free R^2^ = 0.95 were set as the soft-threshold parameters to ensure a signed scale-free coexpression gene network (17). The identified genes in related gene clusters were subjected to gene ontology (GO) and Kyoto Encyclopedia of Genes and Genomes (KEGG) analysis with the “clusterProfiler” package (18) in R software to elucidate the potential mechanisms.

Gene set enrichment analysis (GSEA), performed using the Molecular Signatures Database (MSigDB) (19), was also applied to identify the gene sets that were significantly enriched between tumor samples with high and low IPS scores. One thousand random sample permutations were applied in the analysis.

### IHC and Multiplexed Immunofluorescence Study

The canonical M2 macrophage marker protein CD163 (20) and EMT phenotype markers E-cadherin and vimentin were detected by IHC staining in formalin-fixed, paraffin-embedded (FFPE) tumor specimens from 116 consecutive ESCC patients in our center. IHC staining and IHC score calculation were performed as previously described (21). Briefly, staining was conducted following the immunoperoxidase staining method. Anti-CD163 (clone 10D6, Zhongshan Goldenbridge Biotechnology Co., Ltd., Beijing, China) was diluted 1:100. Both anti-E-cadherin and anti-vimentin antibodies (Cell Signaling Technology Co., Ltd., Danvers, MA, USA) were diluted 1:1000. All IHC slides were examined by two independent pathologists who were blinded to the patients’ clinical information. Three slide fields (×100) were randomly captured. Captured images were processed by using Image-Pro Plus image-processing software (Version 6.0, Media Cybernetics, Inc.) to aid in cell counting and staining density evaluation. Appropriate positive (tumor samples from tissue bank of our center that had previously been shown to express high level of proteins respectively) and negative (substitution of the primary antibody by non-immune immunoglobulin) controls were included in each batch run.

Multiplexed immunofluorescence was performed in some of the typical tumor samples indicated by the IHC staining study. The multiplexed immunofluorescence was performed as previously described (22). Briefly, slides were made using 4-μm thickness of the tumor samples. After pretreatment, relevant primary antibodies targeting CD163, E-cadherin, and vimentin (as described above) were incubated for 2 h at room temperature, then followed by the secondary antibodies and control vector for 20 min. After multiplexing treatment, DAPI (Sigma, D9542) was used to stain the nucleus. The slides were scanned by Vectra 3 high-throughput multiplexed biomarker imaging system (Perkin Elmer).

This study was approved by the Clinical Research Ethics Committee of Nanjing Medical University (2019051). The procedures involving human subjects were in accordance with the Declaration of Helsinki. Based on the retrospective nature and censoring of personally identifiable information in this study, informed consent was waived.

### Statistical Analysis

The chi-square test or Fisher’s exact test was used for categorical variables. The t-test or Wilcoxon rank-sum test was used for continuous variables. Kaplan-Meier analysis was used to estimate survival. The log-rank test was used to compare survival between subgroups. Cox proportional hazard regression was used to perform univariate and multivariate analysis. Variables were subjected to multivariate analysis by a stepwise backward elimination procedure using a threshold P-value of < 0.05. For survival interaction analyses, we constructed a multivariate Cox proportional hazard model to compute the hazard ratio (HR) according to the IPS score and potential clinical and pathological factors related to clinical outcome (23). Analysis of the correlation between M2 macrophages and other immune cells in ESCC was performed using the Spearman correlation method by applying the “corrplot” package in R. A two-sided p-value of less than 0.05 was considered to indicate statistical significance.

## Results

### Baseline Clinical Characteristics of the Enrolled Cohorts

A total of 390 ESCC patients were included in this study. We set the 179 patients in the GEO cohort as the training set due to its larger number of cases. The 95 patients in the TCGA cohort were used as the independent validation set. The 116 patients with ESCC who underwent curative surgery between 2011 and 2013 in our institution (Jiangsu Cancer Hospital, Nanjing, China) were included in the IHC cohort. The mean follow-up times were 60.4, 21.3, and 58.2 months in the training, validation, and IHC cohorts, respectively. The vast majority of patients with ESCC in this study were male. Patients mainly had tumors in tumor node metastasis (TNM) stage II and III. The detailed clinical characteristics of the three independent cohorts of ESCC patients are summarized in **Supplementary Table S1**.

### Derivation of the IPS From the Training GEO Set and Validation in the TCGA Set

**Figure 1:**
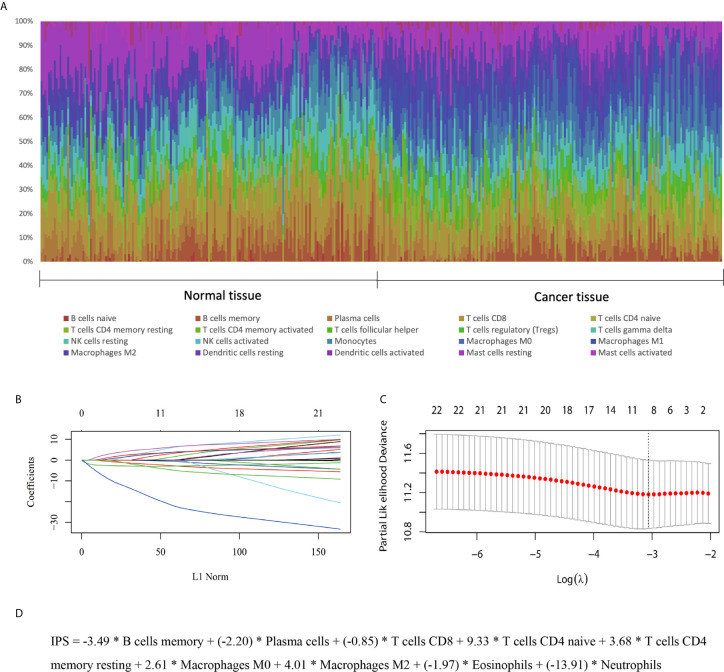
Development of an IPS for ESCC. **(A)** Distribution of 22 infiltrated immune cells in 179 tumor tissues and paired normal tissues of ESCC patients in the GEO training set. Each bar represents the relative proportion of infiltrated immune cells of one tissue. **(B, C)** Tuning of the parameter λ and determination of the coefficients of corresponding immune cell types in the IPS using the LASSO method. **(D)** The derived formula for the IPS.

**Figure 2:**
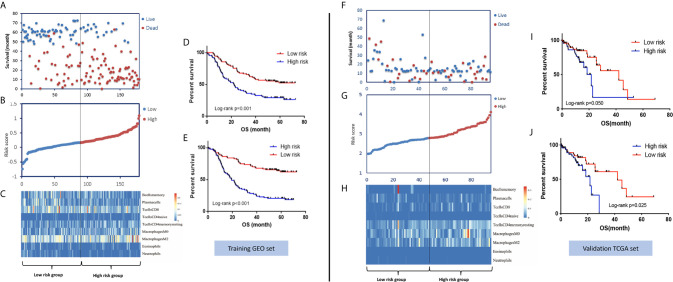
Distribution of risk stratification based on the IPS model and the survival prediction performance of the IPS in the GEO training set and TCGA validation set. **(A)** The distribution of survival status and survival time, **(B)** ranked IPS scores, and **(C)** heatmap of the abundances of the nine immune cell types involved in the IPS model in the GEO training set. Kaplan-Meier survival curves of patients with low- and high-risk scores using the IPS model **(D)** and IPS-TNM model **(E)** in the GEO training set. **(F–J)** Corresponding figures for the TCGA validation set.

In the training set, the abundances of 22 tumor-infiltrating immune cells for each sample were derived using the CIBERSORT algorithm, as shown in **Figure 1A** and **Supplementary Figure S1**. Then, using the LASSO regression method, an IPS, which comprised 9 of the 22 types of immune cells, was constructed. IPS score was calculated as follows: IPS = −3.49 * memory B cells + (−2.20) * plasma cells + (−0.85) * T cells CD8 + 9.33 * CD4-naive T cells + 3.68 * resting memory CD4 T cells + 2.61 * M0 macrophages + 4.01 * M2 macrophages + (−1.97) * eosinophils + (−13.91) * neutrophils, as shown in **Figures 1B–D**. In this model, a higher IPS score was correlated with a higher risk of death. Cell types in the IPS with positive coefficients were positively correlated with a poor prognosis, and those with negative coefficients were positively correlated with a favorable prognosis. Accordingly, patients were ranked into high- and low-risk groups according to IPS score with the median value as the cutoff point. Kaplan-Meier analysis showed that patients with high IPS scores had worse survival than patients with low IPS scores. Additionally, as TNM stage was a robustly established prognostic factor, we found that combining IPS with TNM stage led to more accurate predictive performance, as shown in **Figure 2** and **Supplementary Table S2**.

To validate the survival-predicting performance of the IPS constructed in the training set, the IPS score was calculated for each of the 95 samples in the TCGA validation set. Patients were then ranked into high- and low-risk groups according to IPS score using the median value as the cutoff point. Kaplan-Meier survival analysis validated the prognosis-predicting effect of the IPS, as patients with high IPS scores had significantly worse survival than those with low scores. Again, combining the TNM stage with the IPS score showed a better risk-differentiating capability, as shown in **Figure 2**.

### Robustness Analysis of the Prognosis—Predicting Effect of the IPS in ESCC

To assess the robustness of the predictive performance of the IPS in ESCC, univariate and multivariate Cox regression analyses and survival analyses were performed to assess its relationship with clinical-pathological characteristics (23). The univariate and multivariate Cox regression analyses for the training set and validation set are presented in **Table 1**. The results for both the training set and validation set confirmed that IPS score was an independent prognostic factor for ESCC. Survival analyses of the relationship between IPS and other variables showed that the prognostic effect of the IPS was not significantly modified by any listed variables, as shown in **Supplementary Figure S2**. Our results proved the strong robustness of the IPS as an independent prognostic signature for ESCC.

**Table 1:** Univariate and multivariate Cox regression analyses for overall survival in the GEO training set and TCGA validation set.

Variables	Training GEO set					Validation TCGA set				
	n*	Univariate		Multivariate		n*	Univariate		Multivariate	
		HR (95% CI)	p-value	HR (95% CI)	p-value		HR (95% CI)	p-value	HR (95% CI)	p-value
**Age**										
<60	91	1.00 (reference)		1.00 (reference)		57	1.00 (reference)			
≥60	88	1.55 (1.07–2.31)	0.021	1.51 (1.03–2.22)	0.034	38	1.26 (0.52–3.05)	0.604		
**Sex**										
male	146	1.00 (reference)				81	1.00 (reference)			
female	33	1.28 (0.80–2.04)	0.307			14	0.26 (0.04–1.94)	0.189		
**TNM stage**										
I&II	87	1.00 (reference)		1.00 (reference)		56	1.00 (reference)		1.00 (reference)	
III&IV	92	2.16 (1.45–3.21)	0.001	1.97 (1.32–2.94)	0.003	39	2.51 (1.03–6.15)	0.044	3.03 (1.22–7.50)	0.017
**Differentiation grade**		0.059					0.944		
well	32	1.00 (reference)				16	1.00 (reference)			
moderate	98	1.01 (0.59–1.75)	0.961			48	1.20 (0.33–4.37)	0.781		
poor	49	1.65 (0.93–2.96)	0.090			21	1.03 (0.23–4.61)	0.968		
**Tumor location**			0.257					0.803		
low	62	1.00 (reference)				44	1.00 (reference)			
middle	97	1.14 (0.74–1.74)	0.561			44	0.74 (0.31–1.79)	0.508		
up	20	1.67 (0.91–3.08)	0.101			6	NA			
**Adjuvant therapy**									
no	45	1.00 (reference)				66	1.00 (reference)			
yes	104	1.36 (0.82–2.26)	0.229			25	0.67 (0.22–2.04)	0.484		
**IPS**										
low	90	1.00 (reference)		1.00 (reference)		47	1.00 (reference)		1.00 (reference)	
high	89	2.06 (1.39–3.05)	0.000	2.04 (1.36–3.05)	0.001	48	2.10 (1.01–4.40)	0.049	2.01 (0.91–4.42)	0.083

*Sum of subgroups may not equal to the total sample size due to missing or unknown values.

IPS, immune cell-based prognostic signature; CI, confidence interval; HR, hazard ratio; NA, not applicable.

### The EMT Process Was Associated With the IPS in ESCC

**Figure 3:**
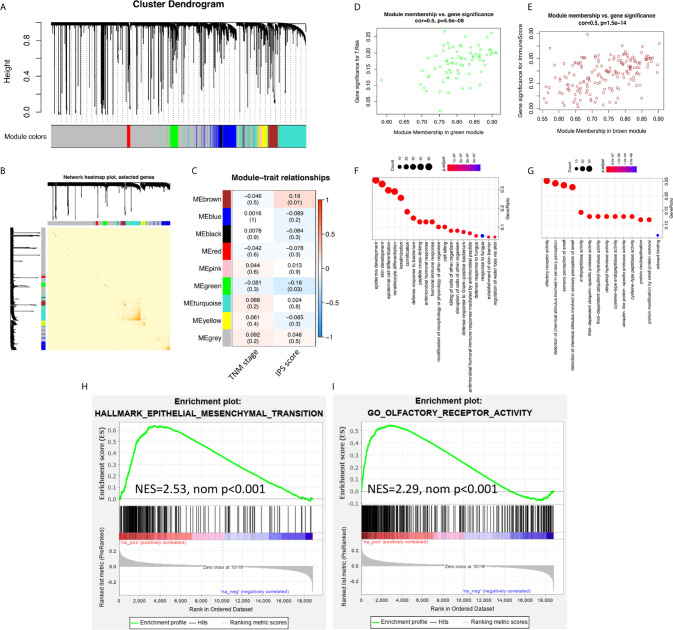
EMT processes were identified in the mechanistic study of the survival-predictive effect of the IPS. **(A)** Nine gene coexpression modules were identified by unsupervised clustering in the WGCNA. **(B)** Interaction relationship analysis of coexpressed genes showed a high-scale independence degree among the nine identified modules in the WGCNA. **(C)** Heatmap of the correlation between module eigengenes and the disease TNM stage and IPS score. **(D, E)** Scatter plots of module eigengenes in the green and brown modules. **(F, G)** Results for the GO annotation of genes in green and brown modules. **(H, I)** GSEA confirmed that pathways related to the EMT process were enriched in green module genes, and olfactory receptor activity pathways were enriched in brown module genes.

To study the inner mechanisms by which the IPS affects the survival of ESCC patients, two different methods were applied independently. The results of the WGCNA are shown in **Figure 3**. In module-trait relationship analysis, the brown module was significantly positively correlated with the IPS score, while the green module was significantly negatively correlated with the IPS score. According to GO and KEGG annotation analyses, the most significantly enriched GO terms of the genes in the brown module were mainly related to epithelial cell differentiation and development. The genes from the green module were mainly related to olfactory receptor activity. The GSEA results are also presented in **Figure 3**. The GSEA results further confirmed the enriched terms of the WGCNA and showed that the immune risk signature/IPS was significantly associated with the EMT process in ESCC. In the mechanistic study, we found that the immune risk signature/IPS was significantly associated with the EMT phenotype in ESCC.

### M2 Macrophages were Identified as the Only Immune Cell Type in the IPS Model that were Significantly Correlated with the EMT Phenotype

**Figure 4:**
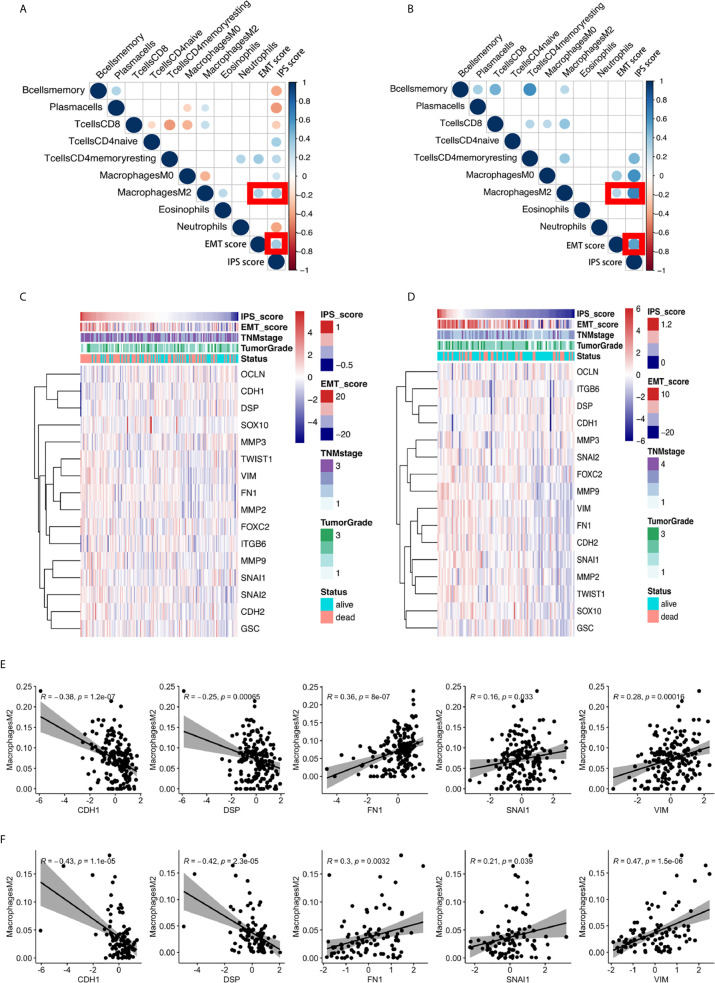
The significant positive relationship between the IPS score and the EMT score and the critical role of M2 macrophages in the relationship between the IPS and the EMT process. **(A, B)** Correlation matrix plots of the correlations between the IPS score, EMT score, and the nine constituent cell types in the IPS in the GEO training set and TCGA validation set. The color of the circle represents the R-squared value of the Spearman correlation analysis. The size of the circle represents the p-value of the correlation; a larger circle represents a smaller p-value, and vice versa. All p-values > 0.001 were filtered from the plots. **(C, D)** Heatmaps of the expression of sixteen canonical EMT genes, IPS scores, EMT scores, and relative clinicopathological features in the GEO training set and TCGA validation set. **(E, F)** The correlations between M2 macrophage abundance and the IPS score, EMT score, and expression of 16 canonical EMT genes in the GEO training set and TCGA validation set.

We further comprehensively investigated the relationship between the IPS score and EMT phenotype. We found a significant positive relationship between the IPS score and the EMT score, a quantitative indicator of the EMT phenotype (5, 24), in ESCC, as shown in **Figures 4A**, **B**. Heatmaps of the IPS score, EMT score (24), related clinicopathological features, and 16 canonical EMT genes (24) are presented in **Figures 4C**, ** D**. The results from the TCGA validation set independently confirmed the findings.

We scrutinized all nine types of immune cells in the IPS model to identify the possible immune cell types dominating the interplay between immune features and the EMT phenotype. After comprehensive correlation analyses, we found that M2 macrophages were the only cell type significantly correlated with the IPS score and EMT score in both the training and validation sets. We further investigated the relationship between M2 macrophage abundance and the gene expression of 16 canonical EMT genes in ESCC. We found that the abundance of M2 macrophages was negatively correlated with the expression of EMT canonical genes CDH1 and DSP and positively correlated with the expression of FN1, VIM, and SNAI1 in both the GEO training set and the TCGA validation set. The results are presented in **Figures 4E**, **F**. For the first time, our results showed the possible critical role of M2 macrophages in the interplay between immune features and the EMT process in ESCC.

### IHC and Multiplexed Immunofluorescence Analysis of ESCC Samples Confirmed the Close Relationship Between M2 Macrophages and the EMT Phenotype

**Figure 5:**
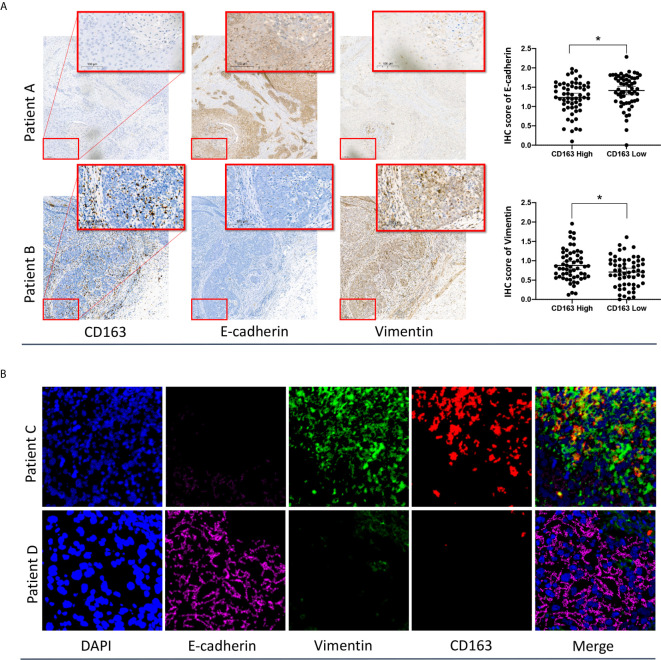
IHC **(A)** and multiplexed immunofluorescence **(B)** analysis of ESCC samples confirmed the close relationship between M2 macrophages and the EMT phenotype. *p < 0.05 according to a t-test.

To validate the relationship between M2 macrophages and the EMT phenotype, IHC staining and Multiplexed immunofluorescence analysis of formalin-fixed, paraffin-embedded ESCC specimens were performed. The basic clinical characteristics of the enrolled IHC cohort are presented in **Supplementary Table S1**. Representative images of IHC staining for CD163, E-cadherin, and vimentin in two individual patients with high- and low-density CD163-positive cells are presented in **Figure 5A**. The IHC results showed that the abundance of CD163-positive cells (M2 macrophages) varied significantly among ESCC specimens. Applying the median CD163-positive cell density as the cutoff value, we categorized the tumors into high and low CD163 groups. We found that the IHC score of the EMT marker E-cadherin in the high CD163 group was significantly lower than that in the low CD163 group. Conversely, the IHC score of vimentin in the high CD163 group was significantly higher than that in the low CD163 group.

The reprehensive images of multiplexed immunofluorescence were presented in **Figure 5B**. The results of our multiplexed immunofluorescence study confirmed that M2 macrophages are significantly correlated with the EMT phenotype in ESCC. The role of M2 macrophages in regulating the EMT process and affecting the prognosis of ESCC deserves further study.

## Discussion

The “seed and soil” theory in malignant tumors, first proposed by Paget in 1989 (25), has been widely recognized and expanded. This theory proposes that the development and progression of tumors depend not only on cytogenetic and epigenetic changes in tumor cells themselves but also on the tumor microenvironment (TME), which contains diverse cell populations, signaling factors, and structural molecules; these components act as the “soil,” supporting the maintenance and growth of the “seeds” in various ways. Recent studies focusing on characterizing the TME in ESCC have provided a glimpse into the vast landscape of factors contributing to the development and progression of ESCC (26–28).

In this study, the abundances of tumor-infiltrating immune cells were derived from gene expression data of ESCC in the GEO training set and TCGA validation set. Then, an IPS to predict the survival of ESCC patients was established by using the LASSO regression method. We applied the median point as the cutoff value, and Kaplan-Meier analysis showed that patients with high IPS scores had a significantly worse overall survival than patients with low IPS scores in both the training and validation sets. The results of univariate and multivariate Cox regression analyses proved that the IPS was a robust prognostic factor for ESCC, independent of age, sex, TNM stage, pathology grade, and tumor location. In the mechanistic study, we performed GSEA and WGCNA separately to determine the underlying pathways and processes by which the IPS components affect the survival of ESCC patients. Our results suggest that EMT, an embryonic program well known for its protumor function, is a crucial component underlying the different prognoses of patients with high and low IPS scores. A higher EMT score was significantly correlated with a higher IPS score, and vice versa. Additionally, the olfactory receptor activity pathway was a significantly misregulated pathway in patients with high IPS risk scores. However, few studies are available about the relationship between the immune phenotype and olfactory receptor activity, which may deserve further research. Further, we scrutinized the roles of all cell components in the IPS in terms of the relationship with the EMT score and IPS score. After systematic correlation analyses, we found that M2 macrophages were the only cell type in the IPS significantly correlated with the EMT score in both the training and validation sets, highlighting the importance of M2 macrophages in the interplay between immune status and the EMT phenotype in ESCC.

In this study, we found a significant relationship between the survival-predicting IPS and the EMT phenotype in ESCC. Although we cannot describe which is the cause or effect, we revealed the interplay between the EMT process and immunity in the TME of ESCC. EMT has been described to have an immune-editing role in the TME of solid tumors. EMT upregulates multiple checkpoint molecules, including PD-L1, which inhibits T cell–mediated cytotoxicity (29), reduces the expression of many adhesion molecules, including E-cadherin, which is a known inhibitory ligand of NK cell receptor (KLRG1) (30) and is linked to the recruitment of macrophages *via* direct regulation of chemokines, such as CCL2 (31, 32). On the other hand, immune cells, including activated CD8+ T cells, macrophages, and several other immune cell types, can produce TGF-β, a strong promoter of the EMT process, regulating the EMT process in solid tumors (33, 34). The results of our systemic correlation analyses highlight the critical role of M2 macrophages in the relationship between IPS and EMT in ESCC. Macrophages exist on a phenotypic spectrum, ranging from M1 type to M2 type: M1 macrophages are “classically” activated macrophages that mainly produce type I proinflammatory cytokines, present antigens, fight infections and have antitumor properties. M2 macrophages mainly produce type II cytokines and have many protumorigenic attributes (35). Our IHC results were similar to those of other previous studies. Jihong and his colleagues showed by the IHC method that M2 macrophages were positively correlated with vimentin but negatively correlated with E-cadherin in ESCC, suggesting that cancer cells may be reprogrammed by M2 macrophages and transformed into cancer cells with more mesenchymal-like properties (36). To our knowledge, M2 macrophages have recently emerged as an increasingly critical immune regulator in the TME of many types of malignancies (37, 38). Several proteins expressed in M2 macrophages, such as SIGLEC10 (39), SIGLEC15 (40), and PD-1 (41), have been newly identified as important therapeutic targets and have been heavily research. The role of M2 macrophages in the prognosis and immunotherapy response of ESCC patients deserves further study.

Although our results showed that M2 macrophages were the only cell type in the IPS that were significantly correlated with EMT score and that they may play a critical role in the TME of ESCC, we found that the survival-predictive effect of M2 macrophages was not as strong as that of the IPS. Patients with high M2 macrophage infiltration showed slightly but not significantly better overall survival than patients with low M2 macrophage infiltration in the GEO training set, TCGA validation set, and IHC data set, as shown in **Supplementary Figure S3**. This may be because M2 macrophages are only one of many constituent immune cells of the TME that affect the survival of ESCC patients. For example, studies have shown that high CD8+ T lymphocyte and CD45+ T lymphocyte infiltration levels facilitate antitumor immunity and are associated with significantly improved survival. High-level B cell and plasma cell infiltration are significantly correlated with an improved prognosis in EC and other cancers (26, 42). Antitumor immunity can be attenuated by other immune cell populations, including regulatory T cells and myeloid-derived suppressor cells, and immune checkpoints such as PD-1. In short, these immune cell populations exert both pro- and antitumor immune effects (3, 12, 43–45) and cooperate in the TME to affect the survival of patients with ESCC.

Several other studies have been published on immune-related prognostic signatures in EC (12, 44–47). However, the present study has some uniqueness and advantages. First, some of the available studies have investigated EC without distinguishing the pathological types, which are mainly ESCC and EAC (12, 44). In fact, the pathophysiology, etiology, and genetic background of ESCC and EAC are very different, and it is more reasonable to treat them separately (48). Second, this study focused on signatures based on tumor-infiltrating immune cells, while others focused on immune-related genes (46). This may be more pertinent to an intuitive understanding of the TME in ESCC. Third, the present study enrolled two independent ESCC data sets; the GEO data set included 179 samples from Chinese patients, and the TCGA data set included 95 samples from multinational patients, representing a more diverse genetic background. Finally, the number of samples included in this study is the largest among the available studies (12, 44–47). The robustness of the results in this study was independently proven in both the training and validation sets, ensuring the high reliability of our results.

There are several limitations to this study. First is the retrospective nature of the study. Although we included two completely independent data sets as training and validation cohorts and also included an IHC cohort to validate our findings, further prospective study is required to validate the robustness of the study’s results. Second, although the present study included the largest number of samples in recent studies, the sample sizes in the training set, validation set, and IHC cohort were relatively small. The study of larger cohorts could further refine our results. Third, although bioinformatic methods are powerful tools for immune cell profiling of the TME, detecting multiple immune cells in tumor samples using multicolor IHC or flow cytometry is still needed to verify our results.

In conclusion, we established a novel nine-immune-cell signature as a prognostic biomarker for ESCC patients. In the mechanistic study, we found a close relationship between EMT and the IPS in the TME of ESCC. Additionally, M2 macrophages were identified as the only cell type significantly involved in the interplay between the IPS and EMT, which may deserve further study.

## Data Availability Statement

The original contributions presented in the study are included in the article/**Supplementary Material**. Further inquiries can be directed to the corresponding authors.

## Ethics Statement

The studies involving human participants were reviewed and approved by The Clinical Research Ethics Committee of Nanjing medical university. Written informed consent for participation was not required for this study in accordance with the national legislation and the institutional requirements.

## Author Contributions

HY and DG analyzed the data and wrote the manuscript. CY and JX contributed constructive suggestion. FY and XH conceived the study. HY and DG contributed equally to this paper. All authors contributed to the article and approved the submitted version.

## Funding

This work was supported by the fund of key project of Jiangsu Cancer Hospital (ZK201607).

## Conflict of Interest

The authors declare that the research was conducted in the absence of any commercial or financial relationships that could be construed as a potential conflict of interest.

## Supplementary Material

Supplementary Figure 1 Violin plot of infiltrated immune cells between tumor tissues and paired normal tissues in the absolute mode of CIBERSORT analysis. The blue color represents the cancer tissues, and the red color represents the paired tumor tissues. The inner violin plot shows the quartile, median, and third quartile.

Supplementary Figure 2 Relationship between the IPS score and overall survival in ESCC. Log(HR) plots of the overall survival rate in the IPS-high and IPS-low groups are shown. Note: *, ** the number of patients in the subgroup was too small, so the result for the log(HR) plots was beyond the −5 to 5 limit.

Supplementary Figure 3 The overall survival-predictive effect of M2 macrophages in the GEO training set **(A)**, the TCGA validation set **(B)**, and the IHC set **(C)**.
