# Supplementary figures and images for: An Immune Cell-Based Signature Associating With EMT Phenotype Predicts Postoperative Overall Survival of ESCC
Source: Front Oncol. 2021 Apr 1;11:636479. doi: 10.3389/fonc.2021.636479 (PMC8047630; doi:10.3389/fonc.2021.636479)

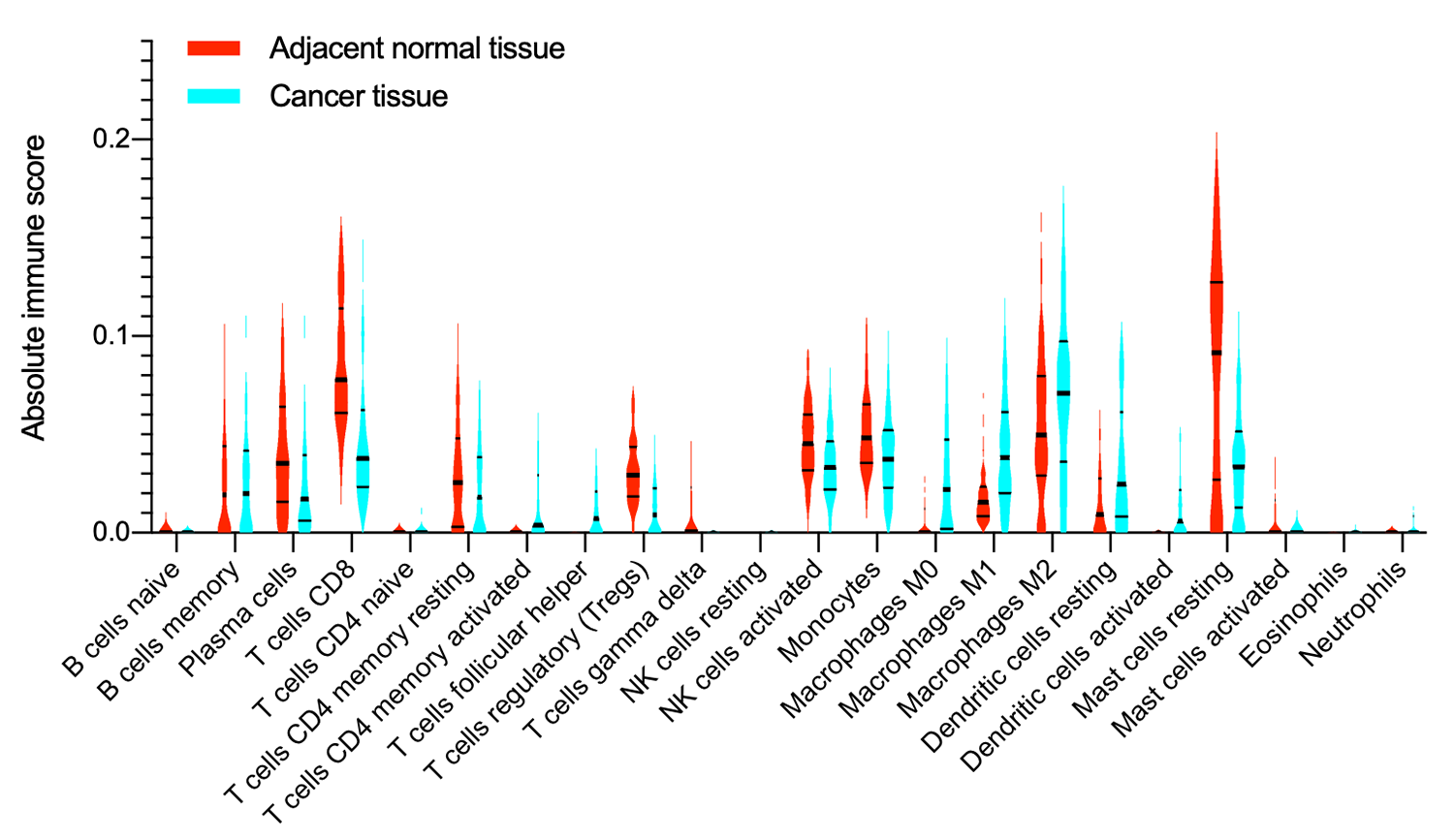

Supplement: Supplementary Figure 1 — Violin plot of infiltrated immune cells between tumor tissues and paired normal tissues in the absolute mode of CIBERSORT analysis. The blue color represents the cancer tissues, and the red color represents the paired tumor tissues. The inner violin plot shows the quartile, median, and third quartile. [file Image_1.tif]

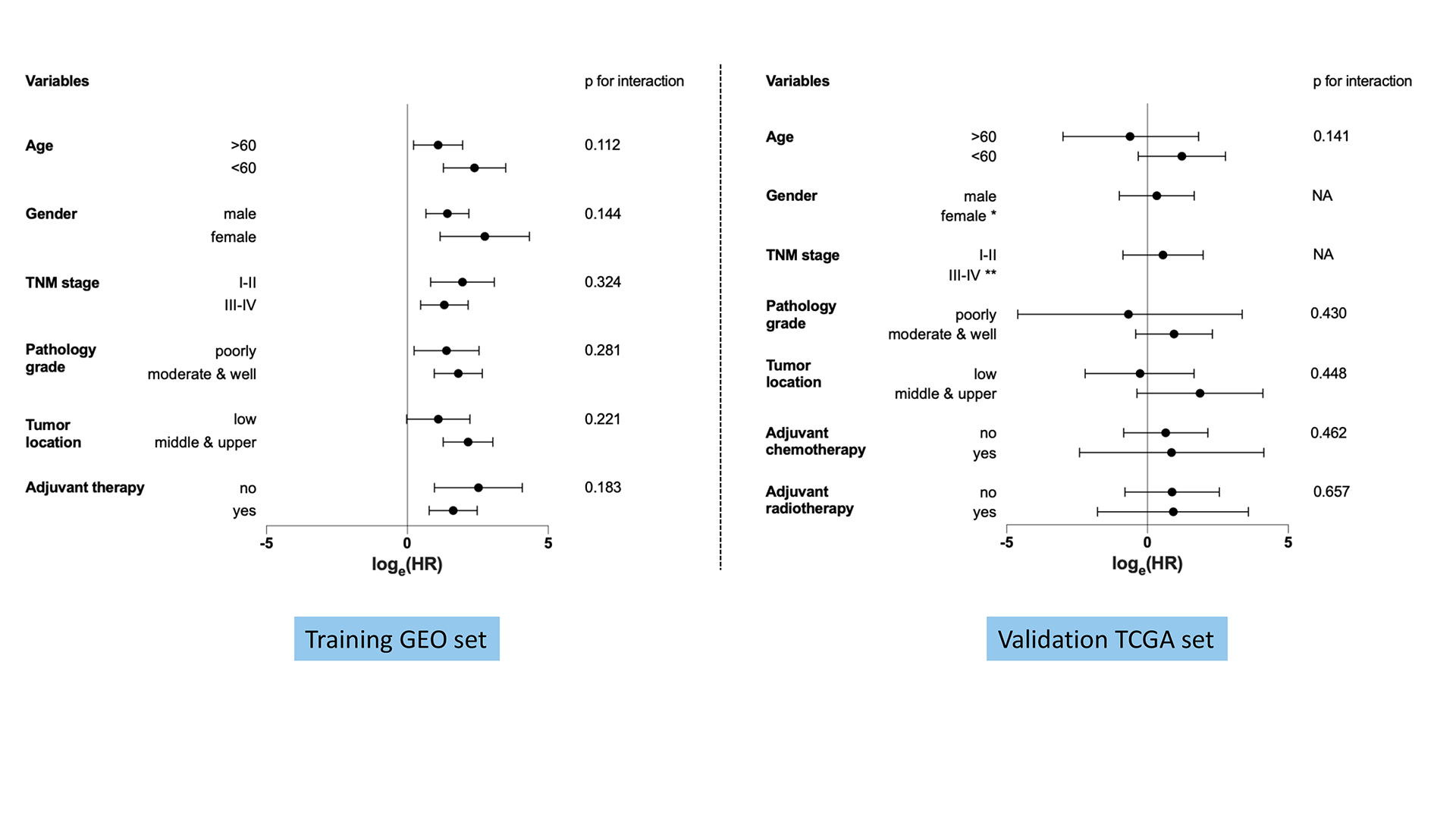

Supplement: Supplementary Figure 2 — Relationship between the IPS score and overall survival in ESCC. Log(HR) plots of the overall survival rate in the IPS-high and IPS-low groups are shown. Note: *, ** the number of patients in the subgroup was too small, so the result for the log(HR) plots was beyond the −5 to 5 limit. [file Image_2.tif]

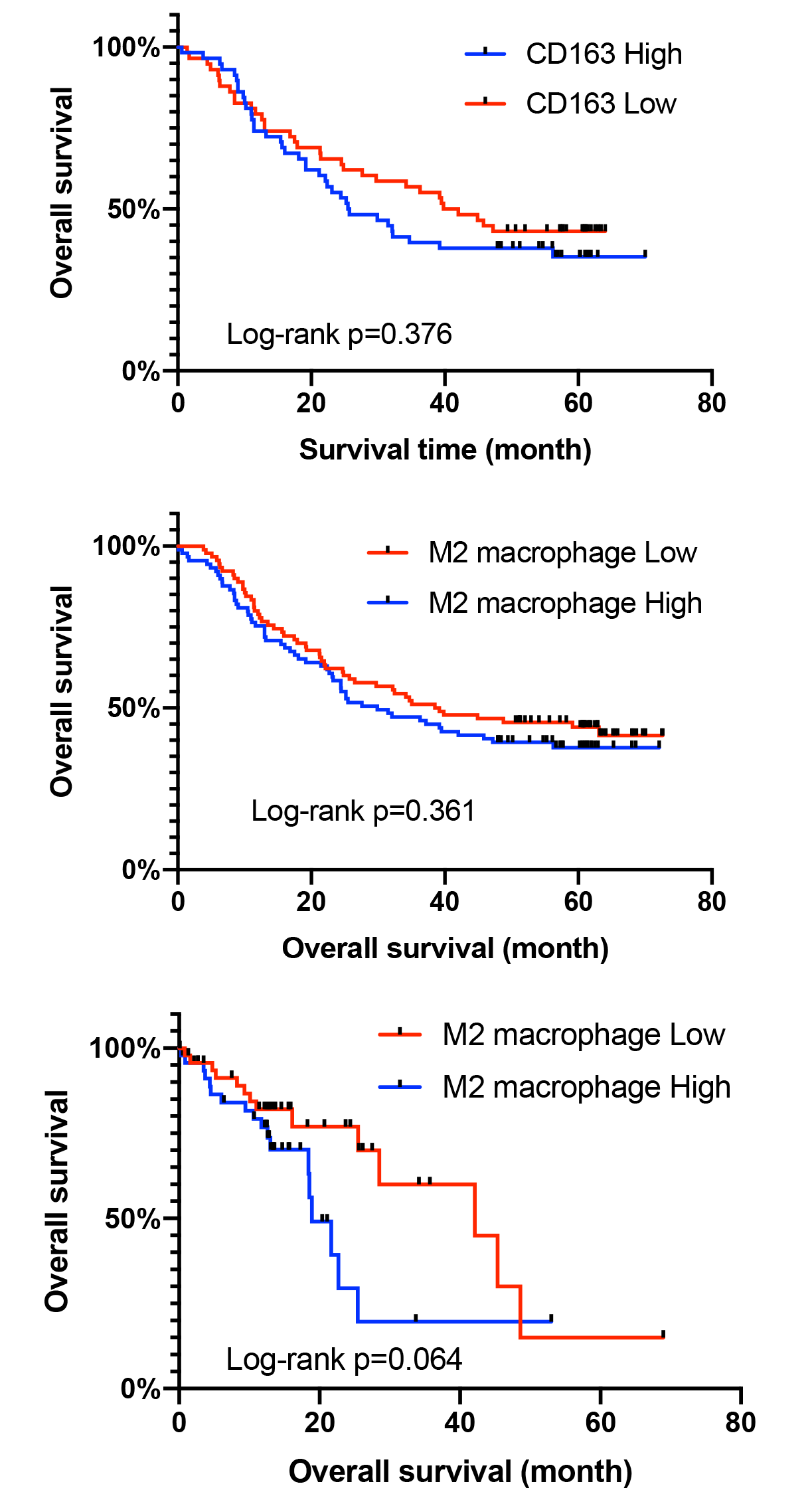

Supplement: Supplementary Figure 3 — The overall survival-predictive effect of M2 macrophages in the GEO training set (A), the TCGA validation set (B), and the IHC set (C). [file Image_3.tif]
